# Optimization and Validation of a Custom-Designed Perfusion Bioreactor for Bone Tissue Engineering: Flow Assessment and Optimal Culture Environmental Conditions

**DOI:** 10.3389/fbioe.2022.811942

**Published:** 2022-03-25

**Authors:** Shuntaro Yamada, Mohammed A. Yassin, Thomas Schwarz, Kamal Mustafa, Jan Hansmann

**Affiliations:** ^1^ Centre of Translational Oral Research, Tissue Engineering Group, Department of Clinical Dentistry, University of Bergen, Bergen, Norway; ^2^ Translational Centre Regenerative Therapies, Fraunhofer Institute for Silicate Research ISC, Würzburg, Germany; ^3^ Chair of Tissue Engineering and Regenerative Medicine, University Hospital Würzburg, Würzburg, Germany; ^4^ Department Electrical Engineering, University of Applied Sciences Würzburg-Schweinfurt, Würzburg, Germany

**Keywords:** perfusion, bioreactor, dynamic cell culture, bone tissue engineering, regenerative medicine, mesenchymal stem cell, shear stress

## Abstract

Various perfusion bioreactor systems have been designed to improve cell culture with three-dimensional porous scaffolds, and there is some evidence that fluid force improves the osteogenic commitment of the progenitors. However, because of the unique design concept and operational configuration of each study, the experimental setups of perfusion bioreactor systems are not always compatible with other systems. To reconcile results from different systems, the thorough optimization and validation of experimental configuration are required in each system. In this study, optimal experimental conditions for a perfusion bioreactor were explored in three steps. First, an *in silico* modeling was performed using a scaffold geometry obtained by microCT and an expedient geometry parameterized with porosity and permeability to assess the accuracy of calculated fluid shear stress and computational time. Then, environmental factors for cell culture were optimized, including the volume of the medium, bubble suppression, and medium evaporation. Further, by combining the findings, it was possible to determine the optimal flow rate at which cell growth was supported while osteogenic differentiation was triggered. Here, we demonstrated that fluid shear stress up to 15 mPa was sufficient to induce osteogenesis, but cell growth was severely impacted by the volume of perfused medium, the presence of air bubbles, and medium evaporation, all of which are common concerns in perfusion bioreactor systems. This study emphasizes the necessity of optimization of experimental variables, which may often be underreported or overlooked, and indicates steps which can be taken to address issues common to perfusion bioreactors for bone tissue engineering.

## Introduction

Critical sized bony defects have only a limited capacity for spontaneous healing. Repair requires extensive surgical intervention using autografts, allografts, xenografts or alloplastic materials (Roddy et al., 2018). However, none of the conventional clinical approaches has achieved the complete repair of native anatomy and function. Tissue engineering approaches, where multipotent cells are combined with scaffold biomaterials to regenerate bone, first emerged in the mid-1980s. Since then, there have been numerous *in vitro* and *in vivo* studies and by 2020, around 150 clinical trials of cell-based bone regenerative therapies had been registered by the U.S. National Library of Medicine (Amini et al., 2012). Mesenchymal stem/stromal cells (MCS) are among the most widely used sources for bone regeneration. MSC are abundantly available from various mesenchymal tissues such as bone marrow, adipose tissue, umbilical cord, and dental tissues (Marquez-Curtis et al., 2015). For scaffolding, three-dimensional (3D) porous scaffolds are preferred, as they mimic the structure of cancellous bone, stimulating MSC towards the osteogenic lineage (Zhang et al., 2018).

A major disadvantage of 3D scaffolds is the low passive diffusion-based mass transport of nutrients and gases, which leads to uneven cell growth within the scaffolds (Rouwkema et al., 2009). To overcome this disparity, various perfusion bioreactor systems have been developed specifically for bone regeneration (Rauh et al., 2011; Yeatts et al., 2013). Perfusion bioreactors provide uniform nutrient supply within the scaffolds while removing waste products, improving cell wellbeing (Rauh et al., 2011). Furthermore, attempts have been made to induce/promote osteogenesis by controlling the mechanical stimulus exerted by fluid flow (Gaspar et al., 2012). In fact, in recent years an increasing number of studies have reported the positive effect of fluid flow on MSC growth and osteogenesis. However, inconsistent results owing to system variation impede a clear understanding of biological responses to the stimuli. This is not only because experimental configuration such as bioreactor design and flow characteristics varies significantly among systems, but also because 3D dynamic cell culture involves various technical challenges which are not encountered in conventional cell culture protocols (Mandenius and Mandenius, 2016). Consequently, each system is to be operated under specific conditions. This is determined by a series of optimization steps: estimation of the magnitude of mechanical stimuli, conditioning of the culture environment and determination of the optimal flow rate for osteogenic differentiation. Nevertheless, environmental conditions applied in dynamic systems seem to be underreported. Indeed, a general caution has recently been issued, noting that a majority of cell culture studies omitted to monitor, control, or report environmental factors such as temperature, gas concentration and medium conditions (Klein et al., 2021).

The first step would be to estimate a promising flow rate by evaluating mechanical stimuli exerted by fluid flow. In the case where a porous geometry is assumed to be isotropic throughout the scaffold, shear stress can be corelated by the Kozeny-Carman equation (Arramon and Nauman, 2001; Daish et al., 2017). However, the equation requires an empirical constant which depends on the geometry of pores, and it may not be suitable for anisotropic and multiphasic porous scaffolds (Truscello et al., 2012). The estimation of fluidics can be alternatively performed by *in silico* modeling, where the flow of culture medium is computationally reproduced in accordance with imported geometry and assigned parameters. In bone tissue engineering, using a scaffold with highly irregular pore geometry and distribution, the high computational cost is a barrier to precise simulation (Zhao et al., 2019). Ideally, a full scaffold geometry acquired by microcomputed tomography (microCT) should be used, but it may not always be feasible because of high computational demand (Jungreuthmayer et al., 2009; Acosta Santamaría et al., 2013). Alternatively, simplified geometry with porous parameters may be employed as a porous medium domain, despite lack of consensus as to its accuracy and predictive power (Campos Marin and Lacroix, 2015). The next step would be to optimize the culture environment. Optimization and validation of each environmental factor are required for successful operation. In conventional culture this may not be given close attention. For example, a perfusion bioreactor often requires a large volume of culture medium to establish continuous flow. The optimal amount depends on the number of vital cells on the scaffolds, and deficiency or excess may cause inhibition of cell growth and paracrine signaling (Schreivogel et al., 2019). Other environmental factors such as temperature, humidity, and static pressure alter MSC behavior, and this needs to be considered in designing systems. A stand-alone bioreactor, namely a bioreactor designed not to be used in a conventional incubator, often consists of complex electric appliances such as various sensors, heating system, gas ejectors, electric outlets and conductors in addition to pump systems (Schuerlein et al., 2017). The installation of these appliances is essential to condition the atmosphere, but highly humid environment is likely to be incompatible with such electrical systems (Rauh et al., 2011; Lane et al., 2014). A major technical challenge is the suppression of air bubble formation, which disturbs fluid flow and damages cells (Sobolewski et al., 2012; Walls et al., 2017; Walsh et al., 2017). Fluidic systems for bone tissue engineering tend to fulfil conditions for bubble formation: highly porous geometry, hydrophobic biomaterials, surfactant in the culture medium, and medium agitation (Sung and Shuler, 2009; Piola et al., 2013; Yu et al., 2017a; Yamada et al., 2021a). All these factors influence cell behavior, and therefore represent uncertainties if not addressed and correctly controlled. Once optimization is completed, the effect of fluid flow on cell behavior may be tested.

The successful operation of perfusion bioreactors for tissue engineering depends largely on the identification and validation of proper culture conditions. Unfortunately, despite an increasing number of studies on 3D dynamic cell culture in bone tissue engineering, comparison of study results is difficult, because each bioreactor system is operated with a unique experimental configuration. Nevertheless, most experimental issues which arise are common to all perfusion bioreactors.

To date, the literature in this field reveals the need for thorough optimization of experimental variables in a perfusion bioreactor. There is also a need to identify and address specific challenges which may arise in using a perfusion bioreactor for 3D dynamic culture. It is important that methods developed to address these issues are readily transferable for application in different bioreactors and can thus serve as general guidelines for designing and setting up flow bioreactor systems. The aim of this study was therefore to identify and optimize experimental variables in a laminar flow bioreactor with an integrated incubation system, by exploring basic cell culture conditions which can be adjusted towards stable dynamic cell culture. The study covers validation of the method applied to estimate fluid effects, the optimization of environmental factors such as medium volume, humidity control, air bubble suppression, and the identification of optimal flow rate. The study was based on rat bone marrow-derived mesenchymal stem/stromal cells (rBMSC), seeded onto 3D polymeric scaffolds for osteogenic differentiation.

## Materials and Methods

### rBMSC Isolation and Expansion

The study was approved by the Norwegian Animal Research Authority (local approval number 20146866) and conducted in compliance with the European Convention for the Protection of Vertebrates used for Scientific Purposes.

rBMSC were isolated as previously described (Yassin et al., 2015). rBMSC from the femurs of Lewis rats were maintained in growth medium consisting of alpha minimum essential medium (α-MEM: 22571-020, Gibco™, United States) supplemented with 1% penicillin and streptomycin (SV30010, HyClone, United States) and 10% fetal bovine serum (FBS: 10270-106, Gibco™, United States) at 37°C in 5% CO_2_ humidified atmosphere. The characterization of rBMSC including the expression of putative MSC markers and the ability of multi-lineage differentiation was previously described (Yamada et al., 2021a). rBMSC from the third to fifth passages were used in the study.

### 3D Porous Scaffold Preparation and Cell Seeding

3D microporous scaffolds (diameter 12 mm, thickness 1.2 mm) were produced by a solvent casting technique as previously described (Odelius et al., 2005; Yamada et al., 2021b). Briefly, a solution of Poly (L-lactide-co-trimethylene carbonate) (LTMC) (RESOMER. LT706 S, Evonik) in chloroform was mixed with sodium chloride (NaCl) particles with a diameter of 90–600 µM in Petri dishes and left with the lids on to allow gradual evaporation of the chloroform. After complete evaporation, the dried constructs were punched into 12 mm pieces and washed thoroughly in distilled water to remove the remaining NaCl particles. The scaffolds were then placed in 48-well plates and exposed to ultraviolet radiation for 2 h, following to washing with 70% ethanol for sterilization. Prior to cell seeding, the scaffolds were pre-wetted in the growth medium for 24 h. 250,000 rBMSC were seeded per scaffold and incubated for 72 h before being transferred into the culture chamber of the bioreactor.

### MicroCT Scanning and Structural Analysis of the Porous Scaffolds

The microstructure of the scaffolds was scanned by microcomputed tomography (microCT) using a voltage of 40 kV and a current of 250 mA at 10 μm spatial resolution (SkyScan 1172: Bruker-MicroCT, Kontich, Belgium). The acquired geometry was exported in *.stl* file for further *in silico* modeling.

### Configuration of the Laminar Flow Bioreactor

**FIGURE 1:**
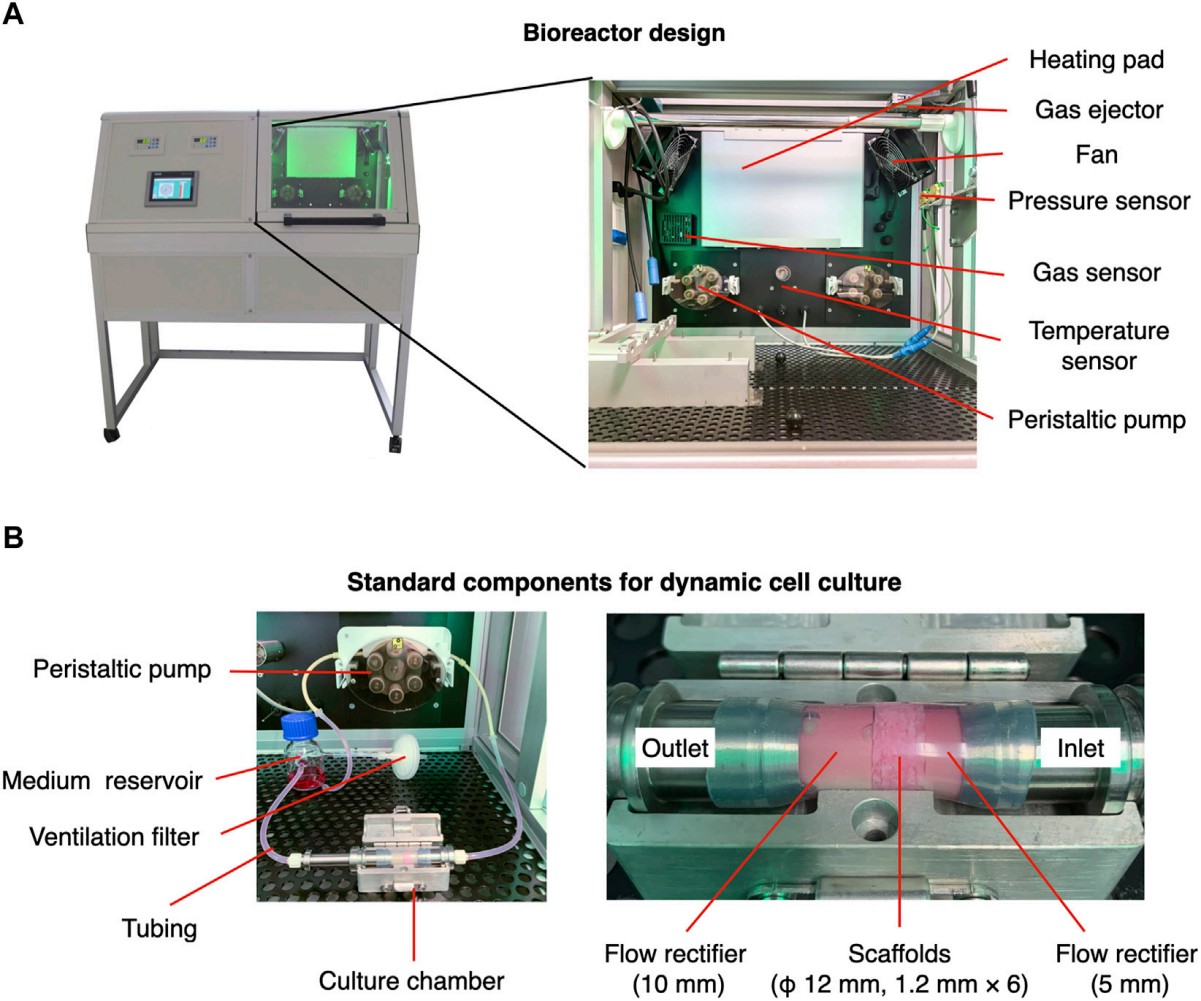
Configuration of perfusion bioreactor used in the present study. **(A)** The bioreactor comprises a controlling/monitoring part (left) and an integrated incubator part (right). The integrated part is designed to maintain the desired temperature and gas concentration for cell culture. **(B)** A medium reservoir and culture chamber where scaffolds are placed are connected by silicon tubes. The flow velocity is controlled by a peristaltic pump.

The laminar flow bioreactor was developed in the Fraunhofer Institute for Silicate Research. It comprises peristaltic pumps and an integrated incubator system, including a heating pad, electric fans, hydropressure sensors, a CO_2_ sensor, and a temperature sensor (Figure 1A). The standard components for dynamic cell culture under perfusion are demonstrated in Figure 1B. The culture chamber, made of stainless steel, was designed to accommodate 3D scaffolds with a maximum diameter of 12 mm. The following sections present the experimental design in detail, including descriptions of specific settings (i.e., tubing, position of medium reservoir, scaffold placement). The perfusion experiment was conducted at 37°C in 5% CO_2_ atmosphere.

### *In silico* Modeling for Fluid Dynamics Simulation

In order to make the microCT data applicable in the simulation, a reference geometry was segmented from the *.stl* file, and a hypothetical whole-scaffold geometry was then reconstructed computationally, by assuming that the segmented geometry represented the microstructure of the scaffold. Precisely, the one fourth of the scanned geometry was repaired and then mirrored to reconstruct a disk-shaped geometry due to a substantial computational burden. Scanning defects such as self-intersections, paper-thin regions, too-narrow edges (i.e., edges smaller than defined minimum element size as mentioned below), and holes were repaired using computer-aided design (CAD) software, MeshLab ver. 2021.05 (Cignoni et al., 2008), Blender ver. 2.92 (Blender, 2021), and Rhino 7 (Robert McNeel & Associates, United States). *In silico* modeling was undertaken in the COMSOL Multiphysics version 5.6 (COMSOL AB, Sweden). Briefly, a simplified geometry was designed with a cylindrical disc with a diameter of 12 mm, which was defined as a porous domain. For the porous domain, porosity was measured by microCT analysis, and permeability was obtained as described previously by measuring pressure drop over cylindrical scaffolds and applying the Darcy’s law (Ramani‐Mohan et al., 2018). The microCT geometry was imported as a solid object. A domain comprising a stack of six scaffolds was then placed in a cylindrical fluid path with a diameter of 12.5 mm, where fully developed flow at either 0.8, 1.6, or 3.2 ml/min was prescribed. It was assumed that fluid flow within scaffolds could be laminar flow because of extremely low flow velocity. Assigned fluid was defined as incompressible Newtonian fluid with the viscosity of water at 37°C (i.e., 0.6913 mPa·s) and density 997 kg/m^3^. Non-slip boundary conditions were enforced at the solid walls. For computation, the geometries were then meshed into linier tetrahedron elements using a physics-controlled mesh module with prescribed mesh resolution “Finer” where the maximum element size, minimum element size, maximum element growth rate, curvature factor, and resolution of narrow regions were defined as 0.429 mm, 0.0464 mm, 1.1, 0.4, 0.9, respectively. Finally, computation was performed by the stationary solver.

A laminar flow was defined by the Navier-Stokes equation as follows:

ρ(u. ∇)u= ∇.[−pI+K]+F

ρ ∇.u=0

$$K=µ\left(\;\nabla u+{\left(\;\nabla u\right)}^{T}\right)$$

where ρ , u , I, p , µ , and F , denote fluid density, fluid velocity, identity vector, pressure, dynamic viscosity, and volume force. For the porous domain used in the simplified geometry, the Darcy flow model was used with Darcy-Brinkman equation as follows:

$$\frac{1}{\varepsilon_{p}}\;\rho \left(u2.\;\nabla \right)u\frac{1}{\varepsilon_{p}}\;=\nabla .\;[-pI+K-\left(µ\kappa^{-1}+\frac{Q_{m}}{\varepsilon_{p}^{2}}\right)u+F$$

ρ ∇.u=Qm

$$K=µ\frac{1}{\varepsilon_{p}}\;\left(\;\nabla u+{\left(\;\nabla u\right)}^{T}\right)-\frac{2}{3}µ\frac{1}{\varepsilon_{p}}\left(\nabla .u\right)I$$

F=0, Qm=0

where $\varepsilon_{p}$ , κ , and Qm denote porosity, permeability, and mass souse. The effect of gravity was not included for simplification. It was modeled by assuming zero mass source and zero volume force due to low fluid viscosity and Reynolds number below 1 (data not shown). Shear stress τ was then computed as previously described using the equation (Egger et al., 2017):

$$\tau =\;µ\frac{\partial µ}{\partial n}$$

where n indicates the x-, y-, and *z*-direction. Shear stress was presented using volume and surface area in the simplified and in the microCT approach, respectively.

### Quantification of Double Strand DNA

Samples were collected in 0.1% Triton X-100, and the cell lysate was obtained by three freeze-thaw cycles. Double strand DNA (dsDNA) was quantified using Quant-iT™ PicoGreen™ dsDNA Assay Kit (P7589, Thermo Fisher Scientific, United States) in accordance with the manufacturer’s protocol. The fluorescence intensity was measured at Ex/Em = 480/520 nm using a microplate reader (VLBL00D0, ThermoFisher Scientific, Finland).

### Quantification of Alkaline Phosphatase Activity

The cell lysate, which was obtained by freeze-thaw cycles, was incubated with P-nitrophenyl phosphate (20-106, Sigma-Aldrich, Germany) for 15 min at room temperature. Absorbance was measured at 405 nm using the microplate reader. ALP activity was normalized by the amount of dsDNA in the samples.

### Immunofluorescent Staining and Confocal Microscopy

Samples were fixed in 4% paraformaldehyde (PFA) for 15 min at room temperature and permeabilized in 0.1% Triton X-100 in PBS (PBSTx) for 15 min at room temperature. Nonspecific binding was blocked with 20% goat serum (G6767, Sigma, United States) in 0.1% Tween-20 in PBS (PBSTw) for 60 min at room temperature. The samples were then incubated with anti-Ki67 monoclonal antibody conjugated with eFluor 660 (50-5698-82, Thermo Fisher Scientific, United States) overnight at 4°C. Filamentous actin (F-actin) and nuclei were counterstained with Phalloidin Alexa Fluor 488 (1:250; A12379, Thermo Fisher Scientific, United States) and 4′,6-diamidino-2-phenylindole (DAPI, 1:5000; 62247, Thermo Fisher Scientific, United States) for 60 min at room temperature, followed by washing five times, for 5 min each, with PBSTw. The samples were mounted in ProLong™ Gold antifade reagent (P36939; Thermo Fisher Scientific, United States). Z-Stack images were acquired by confocal microscopy (TCS SP8; Leica, Germany) and the multichannel images were processed with Fiji/ImageJ (Schindelin et al., 2012). All images in the study were presented as maximum projection z-stack images of 100 µm thickness.

### Live/Dead Staining

For Live/Dead staining, a Live/Dead Cell Viability Kit was used in accordance with the manufacturer’s protocol. Briefly, the samples were washed with Dulbecco’s phosphate-buffered saline (DPBS; 14190-144, Gibco™, United States) 3 times and incubated with 2 µM calcein AM and 4 μM Ethidium homodimer-1 for 30 min at room temperature. The samples were then visualized by confocal microscopy.

### Reverse Transcription Quantitative Polymerase Chain Reaction

Samples for gene expression assay were snap-frozen in liquid nitrogen and stored at −80°C. Total RNA was extracted using a Maxwell^®^ 16 Cell LEV Total RNA Purification Kit (AS1280; Promega, United States) in accordance with the manufacturer’s protocol. Reverse transcription was then undertaken using a High-Capacity cDNA reverse Transcription Kit (4368814; Applied Biosystems, United States). RT-qPCR was performed with the StepOne™ real-time PCR system (4376357, Applied Biosystems, United States) with TaqMan™ Gene Expression Assay (4331182, Thermo Fisher Scientific, United States). The primers used were Runt-related transcription factor 2 (RUNX2, Rn01512298_m1, Thermo Fisher Scientific, United States), Osterix (Rn01761789 m1, Thermo Fisher Scientific, United States), and Glyceraldehyde-3-phosphate dehydrogenase (GAPDH, Rn01749022 g, Thermo Fisher Scientific, United States). The amplification was performed as follows: initial denaturation at 95°C for 20 s followed by 40 cycles at 95°C for 1 s and 60°C for 20 s. Relative gene expression was calculated by the ΔΔCt method, normalized by the endogenous control, GAPDH (Livak and Schmittgen, 2001). The data are presented as a mean value ± standard error (s.e.m) of three replicates.

### Alizarin Red S Staining and Quantification

Samples were fixed in 4% PFA for 40 min and washed three times in Milli-Q^®^ water. The samples were then incubated with 0.1% Alizarin Red S staining (A5533; Sigma-Aldrich, United States) for 20 min at room temperature, followed by thorough washing with Milli-Q^®^ water. For quantification, the dye was extracted in 100 mM cetylpyridium chloride overnight at room temperature. Absorbance was measured at 540 nm using the microplate reader.

### Evaluation of the Effect of Growth Medium Volume on Cell Growth

To evaluate the effect of medium volume on cell growth on a 2D mono-surface and the 3D porous scaffolds, cell growth was evaluated in different volumes of growth medium. For 2D experiments, rBMSC were seeded into wells of 12-well plates at the standard initial seeding density of 5,000 cells/cm^2^. For testing, the cells were separated into three different groups, according to the volume of growth medium: 0.76 ml, the lowest limit of the manufacturer’s recommendation, 1.52 ml, and 3.04 ml, which corresponded to 0.04 µl/cell, 0.08 µl/cells, and 0.16 µl/cells, respectively, at the time of seeding. After 3 and 7 days of incubation at 37°C in 5% CO_2_ humidified atmosphere, the cells were trypsinised, stained with 0.4% Trypan Blue (T10282, Thermo Fisher Scientific, United States), and analyzed by the Countess 2 Automated Cell Counter (Thermo Fisher Scientifics, United States). Likewise, the optimal ratio of cell-to-medium volumes was evaluated on the 3D scaffolds where 250,000 cells were seeded. According to a previous report, the seeding efficiency was estimated to be 50% (Yamada et al., 2021b), and therefore, 125,000 cells were considered to have attached to the scaffold initially. Five scaffolds, corresponding to 625,000 cells, were then incubated with the growth medium at medium-to-cell ratios of either 0.04 µl/cell (i.e., 25 ml), 0.08 µl/cell (i.e., 50 ml), or 0.16 µl/cell (i.e., 100 ml) at 37°C in 5% CO_2_ humidified atmosphere. The medium was refreshed every 72 h. Cell growth was analyzed on days 3 and 7 by quantification of dsDNA.

### Evaluation of the Effect of Humidification on Cell Growth and Viability

To measure medium evaporation during perfusion in the bioreactor, 25 ml of the growth medium was perfused at 0.8 ml/min, 5 ml/min and 10 ml/min at 37°C in 5% CO_2_ environment. During perfusion, the ventilation filter was subjected to either environmental humidity, or humidity enhanced by a water bath (open humidification), or an additional water flask connected to the medium reservoir through a ventilation filter (closed humidification). Gas exchange took place through a ventilation filter attached on the medium reservoir and on the water flask for humidification in the open and closed configuration, respectively. As a control, 25 ml medium was placed statically. Glucose concentration was calculated based on the original concentration and the volume loss of the growth medium. Using growth medium perfused at 10 ml/min for 72 h with and without humidification, rBMSC were incubated at 37°C in 5% CO_2_ humidified atmosphere for a further 72 h in 12-well plates at an initial seeding density of 5,000 cells/cm^2^, or on the scaffolds. Cell growth and viability on the mono-surface were assessed by the Countess 2 Automated Cell Counter (Thermo Fisher Scientifics, United States) and on the scaffolds by Live/Dead staining.

### Evaluation of the Effect of Air Bubbles on Cell Growth and Osteogenic Properties

A dynamic culture system was established by connecting silicon tubes through the medium reservoir, the peristaltic pump, and the culture chamber where six scaffolds with rBMSC were placed. 25 ml of the growth medium was perfused at 0.8 ml/min (i.e., 3 rpm in the system) at 37°C in 5% CO_2_ environment. The medium was refreshed every 72 h. The medium reservoir was either placed at the same level as the culture chamber or approximately 30 cm higher than the chamber, to apply an additional 20 mmHg hydrostatic pressure. After 3 days of perfusion culture, cell growth was evaluated by immunofluorescence with a proliferation marker, Ki67, and quantification of dsDNA.

### Evaluation of Differential Flow Rate and Osteogenic Differentiation

To determine the optimal flow rate for cell growth and osteogenic induction, multiple flow rates were compared. Using the previously determined optimal conditions, the bioreactor was humidified by a water bath and 20 mmHg hydrostatic pressure was applied to the culture chamber. Six scaffolds with rBMSC were placed in the culture chamber, and 25 ml of the growth medium was perfused at 0.8 ml/min (i.e., 3 rpm), 1.6 ml/min (i.e., 6 rpm), or 3.2 ml/min (12 rpm) for 8 h or 24 h per day at 37°C, 5% CO_2_ in a humidified environment. After 7 days of perfusion culture, cell morphology was assessed by immunofluorescence staining. Further analysis was performed on 0.8 ml/min perfusion for 8 h a day. Cell distribution in the first, third, and sixth scaffolds from the inlet was visualized by 0.5% Crystal Violet on day 7. Osteogenic differentiation was assessed by RT-qPCR, Alizarin Red S staining and ALP activity compared to the static culture conditions.

### Statistics

All data are represented as mean ± standard error of mean (SEM) unless stated otherwise (sample size: *n* = 5, except for RT-qPCR *n* = 3). Statistical analysis was performed using SPSS Statistics version 25 (IBM, United States). For pairwise comparison of 2 groups, data were evaluated by Student’s t-test. For multiple comparison, data were evaluated by one-way ANOVA followed by Bonferroni’s multiple comparison test. A *p* value <0.05 was considered statistically significant.

## Results

### Simplified yet Parameterized Geometry as a Substitute for High-Resolution Geometry Obtained From microCT

**FIGURE 2:**
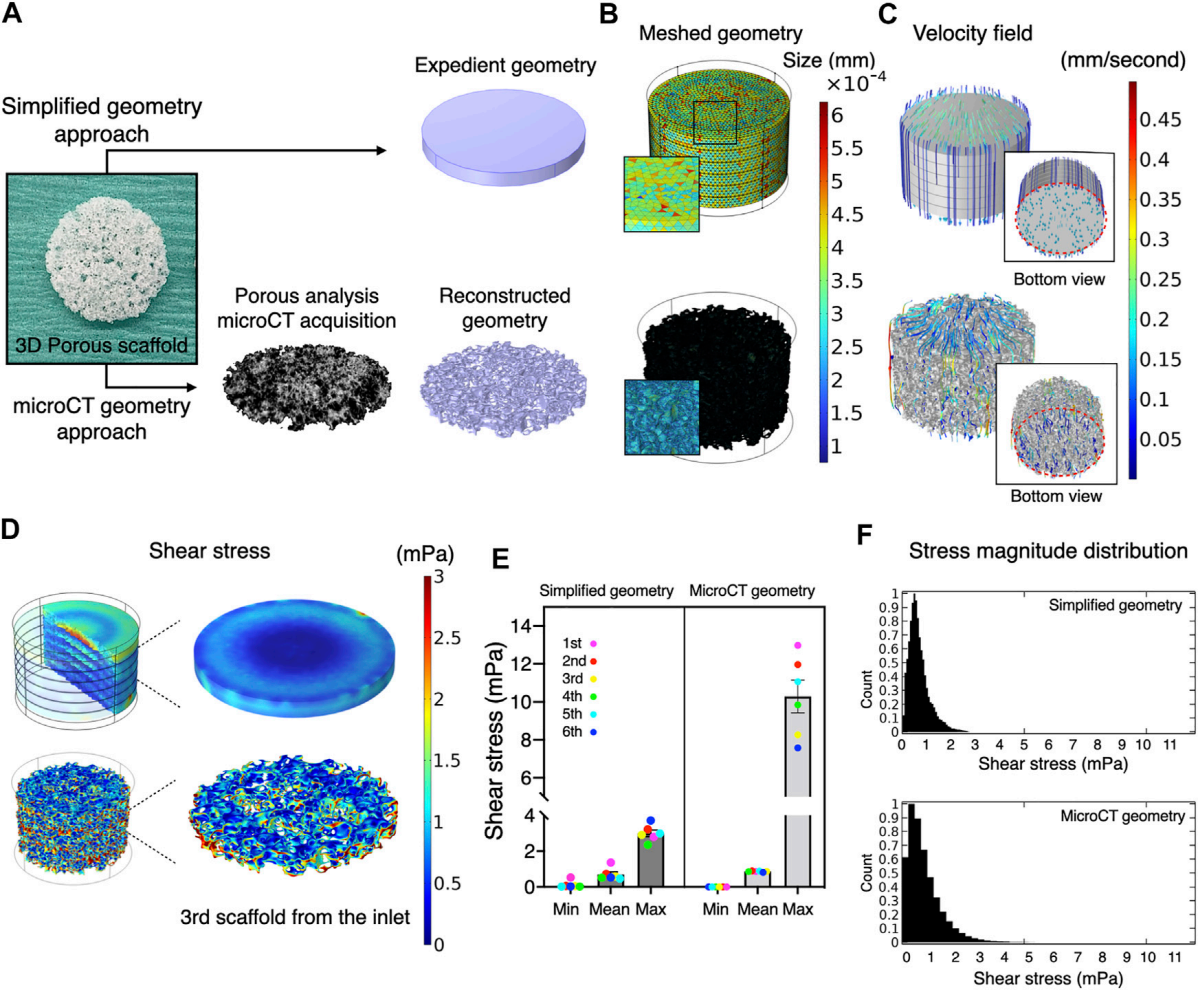
Comparative validation of different modeling methods in fluid dynamics simulation. **(A)** A three-dimensional porous scaffold was modeled by different methods. For the simplified geometry approach, the scaffold was assigned as a porous domain parameterized with porosity and permeability. For the microCT geometry approach, an acellular scaffold was scanned by microCT, and the geometry was imported as a .stl file in CAD software. **(B)** The reconstructed geometry was then meshed for computation. The element size of simplified geometry was significantly smaller than that of the microCT geometry. **(C)** At a flow rate of 0.8 ml/minutes, medium permeated uniformly inside the scaffolds at flow velocities ranging from nearly 0 to 0.5 mm/second in both models. **(D)** Estimated magnitude of shear stress was more patterned in the expedient geometry than in the microCT geometry. The microCT geometry offered more locally specific variation. **(E)** Estimated mean and minimum but not maximum shear stress in the simplified approach was compatible with the microCT approach. **(F)** The distribution of shear stress magnitude shows monophasic peak around 0.5–1 mPa both in the simplified and microCT approaches.

Usually, the magnitude of flow effect on cells is expressed as fluid shear stress. Fluid dynamics simulation was therefore undertaken to estimate shear stress exerted on the scaffold surfaces (i.e., the cells on the scaffold). Conventionally, a simplified geometry parameterized with porosity and permeability is adopted to reduce the computational burden. Therefore, the present study compared such a methodology and whole scaffold geometry, obtained by microCT, to validate predictive power and computational cost (Figure 2A). Despite the same predefined mesh resolution, minimum element size in microCT geometry was notably smaller, and the number of elements comprising the geometry reached nearly one hundred million, while the simplified geometry comprised fewer than 600,000 elements (Figure 2B; Table 1). This resulted in significantly longer free meshing time in the microCT geometry. Likewise, the enormous number of degrees of freedom solved for accounted for substantially longer computation time in the microCT geometry. Both models confirmed that fluid permeated the scaffold domains and showed comparable velocity fields (mean velocity: simplified, 0.15 mm/s; microCT, 0.12 mm/s). Highest flow velocity was estimated along with the wall of fluid column in the microCT model (Figure 2C). This tendency was correlated with the higher shear stress at the peripheral than at the core parts (Figure 2D). In the simplified model, the magnitude of shear stress was more averaged within the scaffold domain. However, the microCT geometry tended to fluctuate more and this was assumed to be due to its geometrical specificity. The estimated values of shear stress varied between the models: the mean and maximum value of shear stress were 0.70 and 3.00 mPa in the simplified geometry and 0.87 and 10.28 mPa in the microCT geometry, respectively (Figure 2E). Despite the difference between the approaches, the frequent distribution of the magnitude of shear stress showed similar trend, converging into the range below 5 mPa (Figure 2F). This suggests that appropriately parameterized geometry may be used to calculate shear stress in an averaged manner but not be suitable for the evaluation of spatial characteristics.

**TABLE 1:** Parameters for meshing and computation in the *in silico* modeling.

		Simplified geometry		microCT geometry
Meshing				
	Predefined mesh resolution		Finer	
	Minimum element size (mm)		0.0464	
	Maximum element size (mm)		0.429	
	Maximum element growth rate		1.1	
	Curvature factor		0.4	
	Resolution of narrow regions		0.9	
	Actual mesh resolution			
	Minimum element size (mm)	0.072428		0.013097
	Maximum element size (mm)	0.4242		0.2669
	Number of element	569570		83848571
	Free meshing time	31 s		1 h 30 min 1 s
Computation				
	Degrees of freedom solved for	203209		45218141
	Computation time	1 min 52 s		28 h 15 min 54 s
	RAM/Processor used	32 GB RAM 2.9 GHz 6 core Intel Core i9		

### Optimization of Medium Volume Required for Optimal Cell Growth in 2D and 3D Culture

**FIGURE 3:**
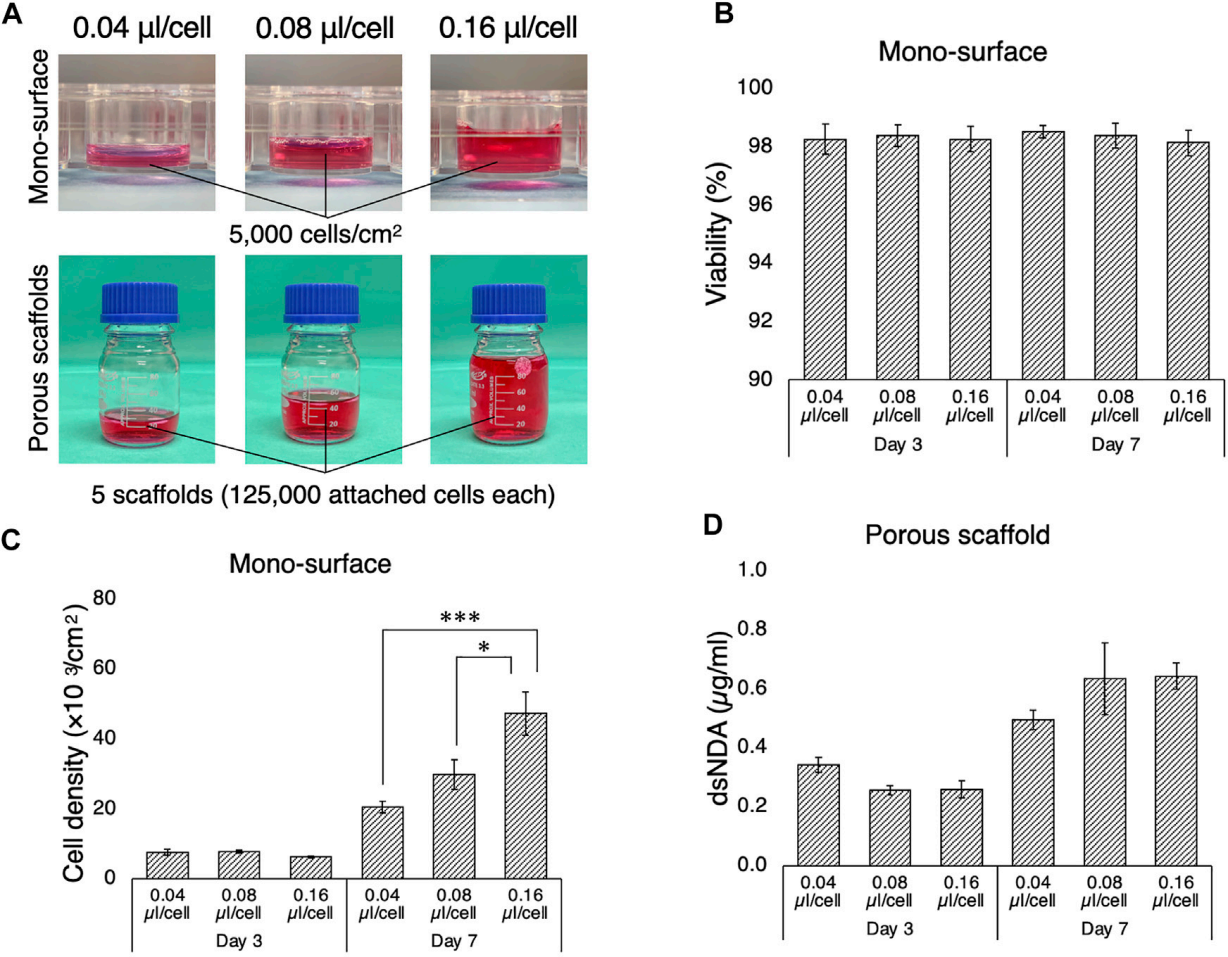
Growth of rBMSC in culture medium at different medium-to-cell ratios. **(A)** Medium-to-cell ratios of 0.04, 0.08, and 0.16 µm/cell at the point of seeding were tested on 2D mono-surface and 3D porous scaffolds. **(B)** Cells seeded on the mono-surface maintained high viability regardless of medium-to-cell ratio. **(C)** On the mono-surface, cell growth was enhanced at a medium-to-cell ratio of 0.04 µl/cell compared to 0.08 and 0.16 µl/cell on day 3, but the tendency was reversed on day 7 when the cell density approached confluence. **(D)** On 3D porous scaffolds, quantification of double-strand DNA (dsDNA) indicated that the medium ratio of 0.04 µl/cell yielded the highest dsDNA on day 3, but medium ratios of 0.08 and 0.16 µl/cell yielded higher dsDNA on day 7. *p < 0.05, ***p < 0.001.

The necessary and sufficient volume of culture medium allows cells to elicit their active kinetics from a paracrine effect while diluting waste products. In most studies, a medium-to-cell ratio is not well described and not standardized. To determine the optimal volume of culture medium for the bioreactor system, rBMSC were cultured for 7 days in different volumes of culture medium, on 2D mono-surfaces and on 3D porous scaffolds (Figure 3A). On the 2D surface, rBMSC maintained high viability of approximately 98% on days 3 and 7, regardless of medium volume (Figure 3B). Cell density on day 3 was slightly lower in the 0.16 µl/cell group, but there was no statistical significance (Figure 3C). On day 7, cell density increased as the medium volume increased, and accelerated cell growth was observed in the 0.08 and 0.16 µl/cell groups (0.04 µl/cell vs. 0.16 µl/cell, *p* = 0.00002; 0.08 µl/cell vs. 0.16 µl/cell, *p* = 0.012). A similar tendency, although not statistically significant, was found in the 3D porous scaffolds (Figure 3D). The lowest medium volume seemed advantageous for initial cell growth by day 3, but the higher medium-to-cell ratios promoted cell proliferation by day 7, when the cells were nearly confluent on the scaffold.

### Minimizing Medium Evaporation During Perfusion Improved Cell Growth and Viability

**FIGURE 4:**
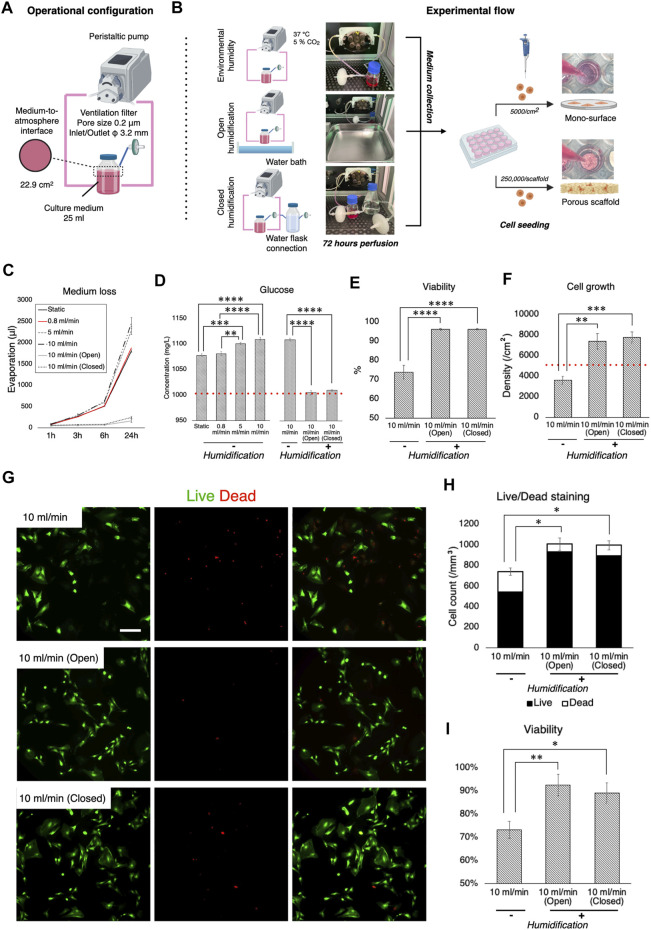
Effect of culture medium loss during perfusion and humidification. **(A)** Operational configuration for the evaluation of medium loss and humidification. **(B)** Illustration of the experimental flow. Culture medium, which was perfused with and without humidification for 72 h, was collected and transferred into cell culture plates where growth of rBMSC on 2D mono-surface and 3D porous scaffolds was observed. **(C)** Evaporation of culture medium occurred in a velocity dependent manner. Humidification with a water bath and a water flask connection effectively prevented evaporation. **(D)** Medium evaporation led to the condensation of medium components (e.g., glucose), disrupting osmotic balance. **(E,F)** On the 2D mono-surface, cell viability and growth of rBMSC incubated in the perfused medium without humidification deteriorated significantly. **(G–I)** Fluorescence images of Live/Dead staining showed that rBMSC incubated in the perfused medium without humidification significantly increased the ratio of dead cells, resulting in low cell density and viability on 3D porous scaffolds. *p < 0.05, **p < 0.01, ***p < 0.001, ****p < 0.0001.

For optimal cell growth, gas exchange is required. Therefore, although perfusion systems are mostly “closed” by tubing, the medium has contact with the atmosphere through ventilation filters, where evaporation takes place. Ideally, the atmosphere needs to be humidified, but most electronic devices such as pumps and sensors are incompatible with high humidity. Therefore, the impact of non-humidified conditions as well as different humidification methods on cell kinetics was evaluated during dynamic culture. Firstly, loss of medium was measured in the bioreactor system during perfusion at different flow rates, with and without humidification, and then the cellular response to perfused medium under such conditions was evaluated (Figures 4A,B). It was confirmed that without humidification nearly 10% of the growth medium evaporated within the first 24 h of perfusion and an amount of medium loss was positively associated with flow rate (Figure 4C). Humidification significantly suppressed evaporation. Notably, using a water bath as the conventional method (open humidification) and connection of water-containing flasks to the ventilation filter on the medium reservoir (closed humidification) reduced evaporation equivalently. Medium evaporation theoretically led to condensation, resulting in elevated content concentration including a glucose level as represented (static vs. open/closed humidification, *p* < 0.0001) (Figure 4D). rBMSC were then incubated under standard culture conditions (i.e., 95% relative humidity, 37°C, 5% CO_2_) in the growth medium, which was perfused at 10 ml/min for 72 h (either with or without humidification) on the 2D surface and the 3D porous scaffolds. On the 2D surface, cell viability was significantly affected in the condensed medium, declining by approximately 20% compared to the humidified environment (without vs. with open/closed humidification, *p* < 0.0001) (Figure 4E). Similarly, cell growth deteriorated in medium perfused without humidification (without vs. with open humidification, *p* = 0.0019; without vs. with closed humidification, *p* = 0.00086) (Figure 4F). On the 3D scaffolds, Live/Dead staining showed that open as well as closed humidification effectively supported cell growth while the condensed medium led to reduced cell growth (without vs. with open humidification, *p* = 0.0041; without vs. with closed humidification, *p* = 0.029) and viability (without vs. with open humidification, *p* = 0.025; without vs. with closed humidification, *p* = 0.011) (Figures 4G–I).

### Suppression of Air Bubbles During Perfusion is a Determinant of Cell Growth and Osteogenic Properties

**FIGURE 5:**
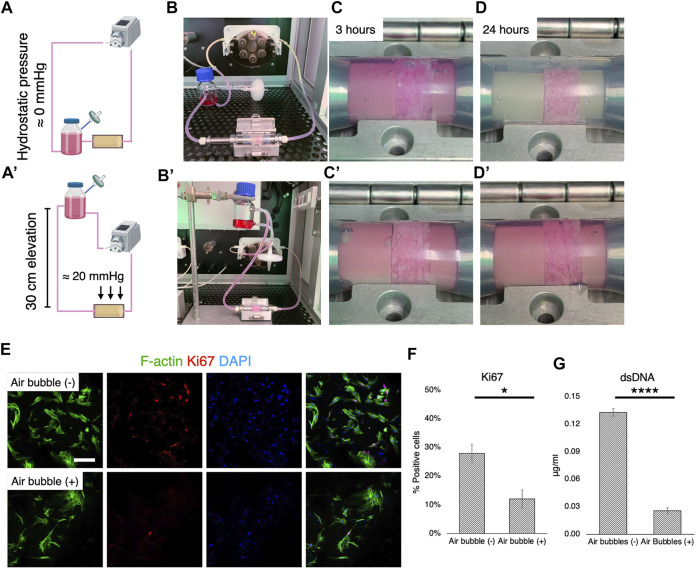
Cell growth deterioration due to air bubbles and suppression. **(A-B, A′-B′)** Schematic illustrations and optical pictures of experimental configuration. The elevation of the medium reservoir by 30 cm corresponded to 20 mmHg hydrostatic pressure. **(C-D, C′-D′)**. With the experimental configuration, air bubbles were vigorously generated, mostly due to hydrophobic porous scaffolds and medium agitation in the scaffold chamber. This was effectively prevented by the application of hydrostatic pressure at 20 mmHg. **(E,F)** Immunofluorescence images and quantification of a proliferation marker, Ki67, showed that cell proliferation was significantly affected under the environment with air bubbles. **(G)** Quantification of double strand DNA confirmed the inhibition of cell growth by air bubbles in the perfusion system. Scale bar: 100 μm. *p < 0.05, ****p < 0.00001.

The formation of air bubbles is an acknowledged problem in perfusion systems (Lochovsky et al., 2012; Kang et al., 2014), and the system used in this study was no exception. Therefore, according to Henry’s law, suppression of air bubble formation was attempted by controlling static pressure (Figures 5A,A′). When the medium reservoir was placed at the same level as the culture chamber, air bubbles were generated rapidly within 3 h of perfusion (Figures 5B,C). After 24 h of perfusion, airspace dominated in the chamber, with the surfaces of fluid paths mostly dried, although the medium permeated the scaffolds with the help of capillary action (Figure 5D). When the reservoir was vertically positioned 30 cm higher than the culture chamber, however, the growth medium filled the chamber space (Figures 5B′–D′). This corresponded to a hydrostatic pressure of approximately 20 mmHg. In the environment where air bubbles were formed, the cells appeared to be less elongated and scattered and the expression of a proliferation marker, Ki67, was significantly downregulated (Figure 5E). Quantification revealed that approximately 30% of rBMSC were proliferative without air bubbles, while only 12% of the population expressed Ki67 (Figure 5F) (*p* = 0.041). The quantification of dsDNA confirmed the adverse effect of air bubble formation on cell growth, significantly suppressing cell proliferation (*p* < 0.0001) (Figure 5G).

### Optimal Flow Rate Triggers Osteogenic Differentiation of rBMSC

**FIGURE 6:**
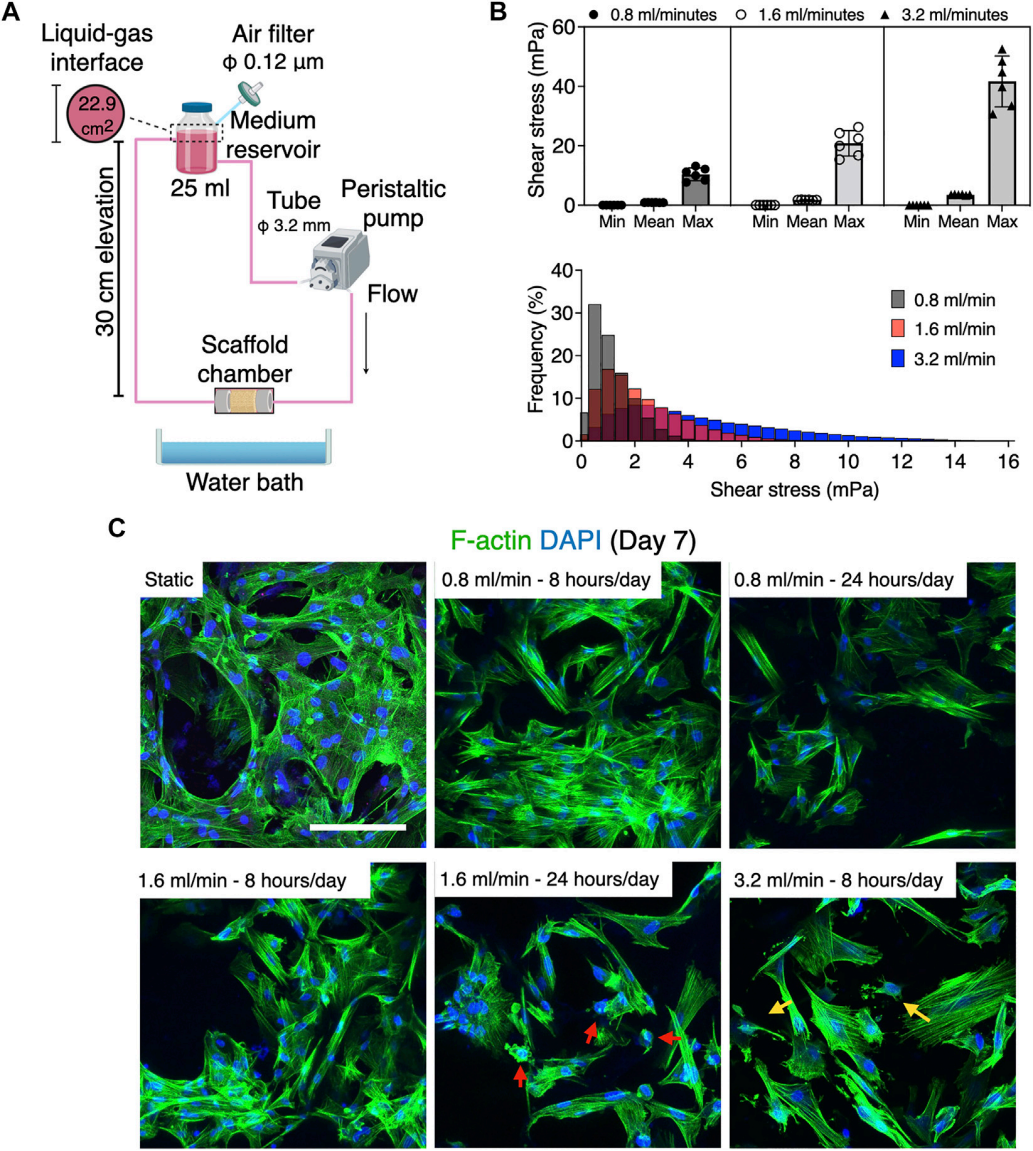
Differential cell response to fluid flow at 0.8, 1.6, and 3.2 ml/min. **(A)** Schematic illustration of experimental configuration. **(B)** Estimation of fluid shear stress by *in silico* modeling using the microCT approach. The medium perfusion at 0.8, 1.6, and 3.2 ml/min in the present system exerted shear stress ranging from nearly 0 to 10 mPa, nearly 0–21 mPa, and nearly 0–41 mPa, respectively. The histogram shows the mode value of shear stress distribution. **(C)** Fluorescence images of rBMSC exposed to fluid flow. While the cells in the static control elongated homogeneously, those under perfusion tended to show more contracted morphology and more filamentous activity. Flow rate at 0.8 and 1.6 ml/min for 8 h supported cell growth, whereas perfusion for 24 h a day inhibited cell proliferation or induced apoptotic response (red arrows). A flow rate of 3.2 ml/min was found to fractionalize the cytoskeleton (yellow arrows). Scale bar: 100 µm.

**FIGURE 7:**
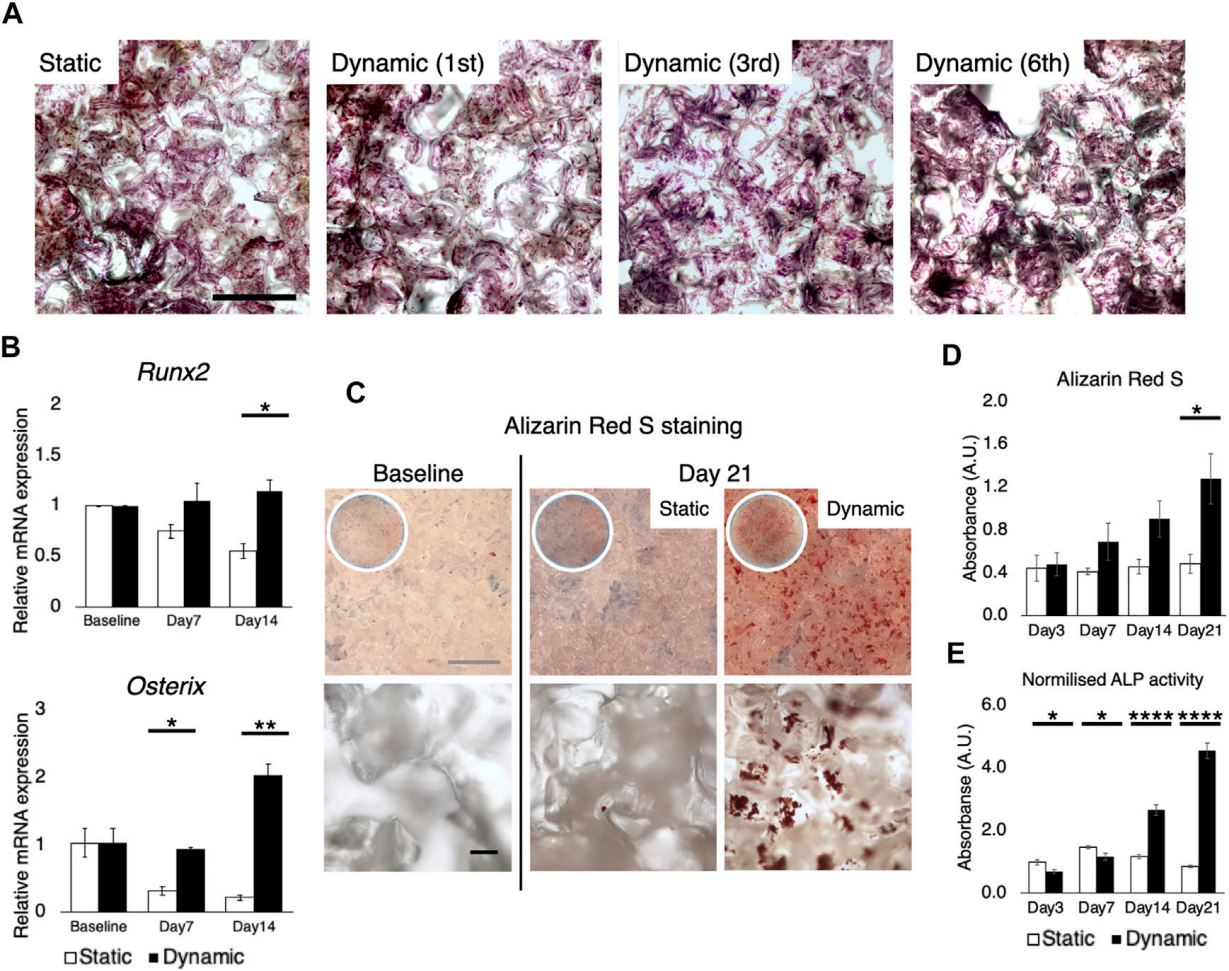
Osteogenic differentiation induced solely by fluid stimuli at 0.8 ml/min. **(A)** Crystal violet staining showed, as expected from the computational simulation, that the cells were uniformly distributed throughout the stack of scaffolds, regardless of the distance from the inlet. Scale bar: 500 µm **(B)** RT-qPCR showed the upregulation of key transcription factors for osteogenesis, RUNX2 and osterix, on days 7 and 14 under perfusion while the cells gradually lost the osteogenic property in the static environment. Baseline stands for 24 h after cell seeding on the scaffold **(C,D)** Alizarin red S staining confirmed the formation of mineralized deposit on the scaffolds subjected to perfusion for 21 days. Scale bar: (grey) 1 mm, (black) 100 µm **(E)** Perfusion culture led to the activation of alkaline phosphatase activity on days 14 and 21. *p < 0.05, **p < 0.01, ***p < 0.001, ****p < 0.0001.

MSC are exquisitely sensitive to mechano-environmental factors. The response varies, depending on the magnitude and duration of fluid stimulation (Maul et al., 2011; Lane et al., 2014). Initially, several flow rates and perfusion time were tested, to determine conditions at which cell growth was optimal: 25 ml of the growth medium was perfused under humidification by a water bath in the bioreactor, wherein 20 mmHg hydrostatic pressure was applied to the culture chamber (Figure 6A). Flow rates of 0.8 ml/min, 1.6 ml/min, and 3.2 ml/min were compared, corresponding to shear stress ranging from nearly 0 to 13.1 mPa (mean 0.88 mPa, mode value 0.5–1 mPa) to 26.2 mPa (mean 1.76 mPa, mode value 1.0–1.5 mPa), and to 52.6 mPa (mean 3.51 mPa, mode value 2–3 mPa), respectively, as estimated by the *in silico* modeling (Figure 6B). Perfusion for 8 h a day at 0.8 ml/min and 1.6 ml/min supported cell growth while 3.2 ml/min caused fragmentation of cytoskeletal structures (Figure 6C). However, perfusion for 24 h was found to suppress cell growth and in particular, perfusion at 1.6 ml/min induced cell damage and apoptotic response. These experiments disclosed that in the present system, perfusion for 8 h at 0.8 ml/min (mean 0.88 mPa, mode value 0.5–1 mPa) provided optimal fluidic stimulus. At the flow magnitude, no noticeable differences in cell distribution were observed among the first, third, and sixth scaffolds from the inlet, which is consistent with the observation by the computational model (Figure 7A). rBMSC subjected to the fluid flow upregulated the key osteogenic transcription factors, RUNX2 and Osterix, on days 7 and 14, while under static conditions the cells gradually lost the osteogenic property (RUNX2, *p* = 0.23; Osterix, *p* = 0.032 on day 7; RUNX2, *p* = 0.049; Osterix, *p* = 0.007 on day 14) (Figure 7B). Alizarin Red S staining confirmed that perfusion culture of rBMSC resulted in calcium deposition, (Figure 7C), which became more pronounced over time (*p* = 0.034 on day 21) (Figure D). This was accompanied by an increase in ALP activity (*p* = 0.019 on day 3; *p* = 0.032 on day 7; *p* < 0.0001 on days 14 and 21) (Figure 7E).

## Discussion

The application of dynamic cell culture shows promise in bone tissue engineering, in which a 3D porous scaffold is a critical component of successful bone regeneration (Gaspar et al., 2012). This is mainly because medium flow homogenizes gas and nutrient concentrations within the scaffolds while removing waste products, preventing the cells in the core part of the structure from succumbing to deprivation of gases and nutrients (Bergemann et al., 2015). Moreover, previous studies on 2D systems have reported that MSC are mechanosensitive, and appropriate fluidic shear stress may direct them towards the osteogenic lineage (Holtorf et al., 2005; Yourek et al., 2010; Kim et al., 2014; Becquart et al., 2016; Stavenschi et al., 2017; Tsai et al., 2019; Dash et al., 2020). With reference to clinical translation, a number of perfusion bioreactor systems have been developed and tested using 3D porous scaffolds (Livak and Schmittgen, 2001; Yourek et al., 2010; Maul et al., 2011; Kang et al., 2014; Kim et al., 2014; Lane et al., 2014; Bergemann et al., 2015; Becquart et al., 2016; Dash et al., 2020). However, due to the complexity of the 3D culture system, each of the systems has unique features and applies original experimental configurations. The conclusions drawn from various studies are therefore inconsistent and sometimes contradictory. This hinders cross-study comparison of different systems and the development of further optimized systems. Therefore, the aim of the present study was to identify and validate inconsistencies, mainly associated with environmental variables and then to optimize experimental configuration in the perfusion bioreactor system for bone tissue engineering.

**FIGURE 8:**
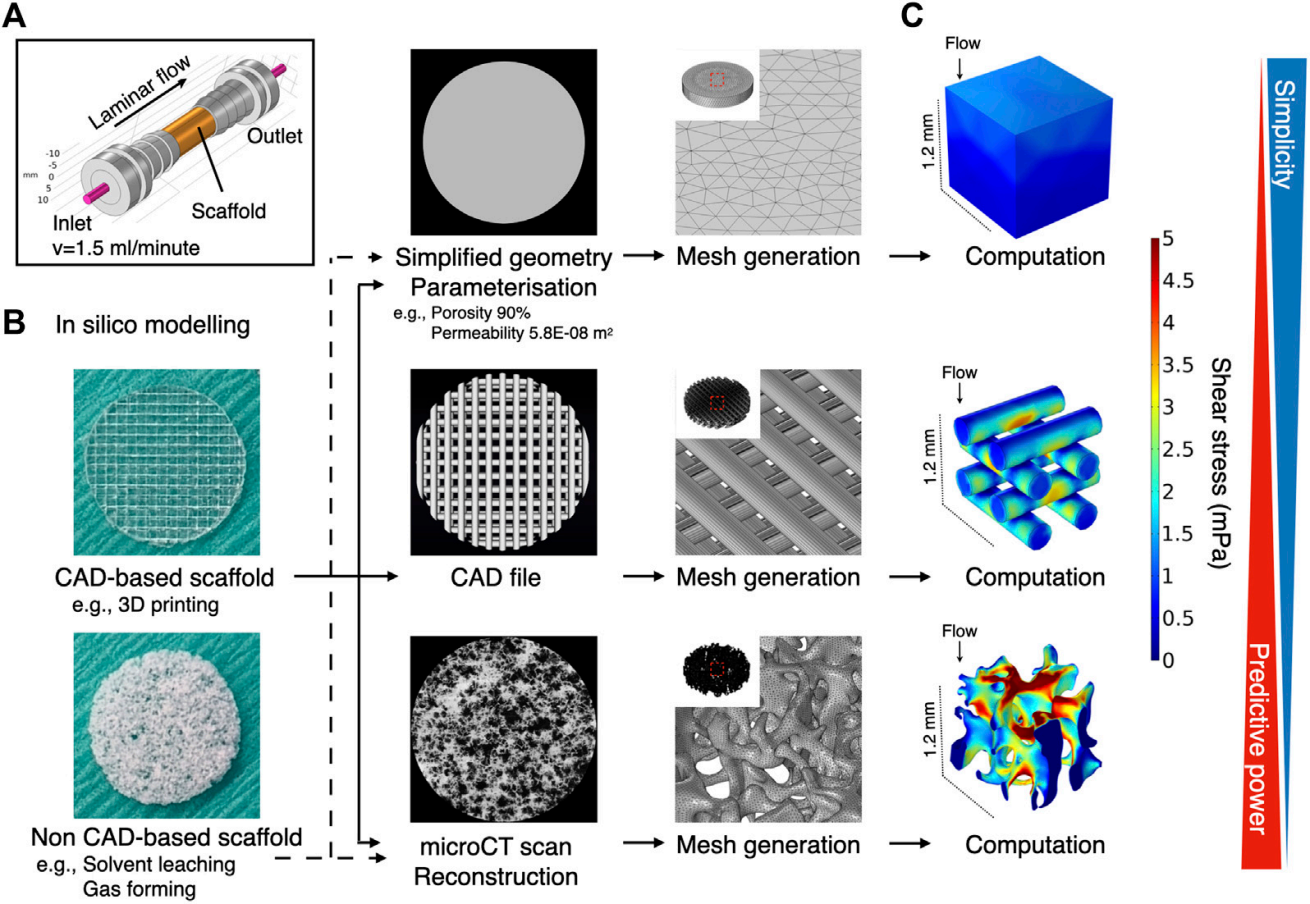
Schematic illustration of common methods for computational fluid dynamic simulation in scaffold-based perfusion cell culture. **(A)** Expedient culture chamber design. Porous scaffolds were placed in the culture chamber and perfused at 1.5 ml/min. **(B)** The computational reproduction of scaffold geometry can be undertaken using different methodologies, depending on the availability of hardware resources and feasibility. At its simplest, expedient geometry (e.g., a cylinder) may be assigned as a porous domain where porous parameters are input. The generated mesh for such a geometry tends to be coarse, being less demanding computationally. Alternatively, CAD data may be used when the scaffold is designed in CAD software (e.g., 3D printed scaffold). The mesh required for computation tends to be finer than the simplified geometry, but regular. The highest predictive power may be expected when microCT data are imported to acquire the actual geometry used in the study. Mesh generation and computation entail high computational time and costs. **(C)** The Darcy (-Brinkman) model may be applied to calculate shear stress for porous domain, whereas laminar flow may be defined by the Navier-Stokes equation by assuming that culture medium is an incompressible Newtonian fluid.

Cell response to fluidic stimuli differs according to the magnitude of shear stress exerted by fluid in motion (Yeatts and Fisher, 2011). Despite the wide application of flow rate (e.g., ml/minute) or pump speed (e.g., rpm) as parameters to describe the characteristics of fluid stimuli, neither represents the magnitude of flow to which the cells respond or can be used to compare the results of different bioreactor systems unless the flow rate/pump speed is correlated with the magnitude of fluid force to the cells by mathematical models or computational simulation (Sladkova and de Peppo, 2014). To reduce uncertainty when comparing study results, the accurate estimation of fluid shear stress serves as a common reference point. The magnitude of shear stress is determined by local velocity. In contrast to 2D experimental settings, where fluid motion is limited to the *X*-*Y* direction, evaluation of fluid shear stress in 3D bioreactor systems presents a major challenge. This is attributable primarily to the geometry of porous scaffolds, in which local velocity varies from one point to another and possibly from moment to moment. Conventionally, the mathematical model applying Kozeny-Carman equation has been used to analyze shear stress within a homogeneous porous domain (Podichetty and Madihally, 2014). For the analysis of spatial shear stress distribution, an *in silico* modeling is a powerful alternative for studying microfluidics in such a complex environment. It allows fluid to be virtually traced within a given geometry and fluid dynamics to be computed. The tool has been applied in some tissue engineering studies, to examine microfluidics in bioreactor systems (Geris et al., 2016). In most cases, *in silico* modeling was performed using a simplified geometry (e.g., a cylinder), idealized by parameterization to reduce the computational burden (Zhao et al., 2019; Ramani‐Mohan et al., 2018; Egger et al., 2017; Nokhbatolfoghahaei et al., 2020; Pereira et al., 2021; Melke et al., 2020). Parameterization with porosity and permeability allows the geometry to be considered as a porous domain to which Darcy flow model may be applied. This approach could be used even if a scaffold consists of several domains as long as each domain possesses a homogeneous structure. Alternatively, a CAD geometry in the case of, e.g., 3D printed scaffolds, or a geometry acquired by microCT may be used as more accurate methods where Navier-Stokes equation may be applied by assuming that a liquid property is defined as an incompressible Newtonian fluid (Figure 8). The present study indicates that the simplified approach may capture the averaged characteristics of fluid dynamics within the porous domains, but it does not resolve the velocity field in detail because the model does not include the geometrical information of pores. Indeed, the velocity within the porous domains is expressed as the Darcy velocity. This indicates that the velocity within the porous domains is expected to be uniform compared to the velocity within the pores in the detailed model, and the gradients associated with the Darcy velocity are likely to be computed smaller than the counterpart. Therefore, the simplified approach can only compute shear stress in an average sense expediently and is not suited for spatial estimation in detail. This highlights the superiority of the microCT approach where actual shear stress within the pores is explicitly resolved. The microCT-based modeling revealed great spatial variations in estimated shear stress. This suggests that cell response within the scaffold constructs is likely to be heterogeneous. In other words, it would be recommendable that biological events in a 3D perfusion system is explained by the range and frequency of shear stress distribution, but not just by the mean. For visualization of local shear stress distribution, microCT geometry is advantageous. This fact also emphasizes that the microCT approach provides possibility to correlate observed cell behavior with a magnitude of shear stress in a single cell resolution, leading to more accurate investigation on dynamic cell culture (Jungreuthmayer et al., 2009). However, the considerably greater computational burden may be a major disadvantage in the case where a complex or large scaffold geometry is to be modeled for computation. In fact, a great amount of time was often required, not only to analyze, but also to repair and reconstruct microCT data to be compatible with CAD and *in silico* modeling software (Acosta Santamaría et al., 2013). It is acknowledged that simulation of fluid dynamics using a full-scale scaffold is not always feasible, depending on available hardware and model complexity (Acosta Santamaría et al., 2013; Zhao et al., 2019). To achieve a balance between predictive visualization and computational cost, segmentation of region of interest (ROI) from the whole scaffold geometry seems a valid procedure for demonstrating representative shear distribution (Lane et al., 2014; Sellgren and Ma, 2015; Daish et al., 2017; Pasini et al., 2019). In short, the simplified method is effective for estimating a range of shear stress with a minimal computational burden when the porous property is properly parameterized, but simulation with microCT geometry is essential to gain insight into local fluctuations of fluid dynamics. Noteworthily, in the study, the simulation was performed using an acellular scaffold, and the values may not necessarily represent later timepoints because of cell growth and deposited extracellular matrix (Ramani‐Mohan et al., 2018; Nokhbatolfoghahaei et al., 2020). Furthermore, it identified the fluid property of culture medium with water at 37°C, and therefore, further investigation is required for culture medium specific dynamics in the perfusion systems.

Next, the volume of culture medium was optimized in the present system. Perfusion bioreactors commonly consist of medium reservoirs, tubes, and culture chambers. These hold unique dimensions specifically adapted to each system and the volume of medium required needs to be modified accordingly. In general, a perfusion bioreactor requires a large volume to establish continuous flow, but the amount needs to be adjusted with reference to the vital cells on the scaffolds. Therefore, cell growth was compared in different medium-to-cell ratios, on 2D mono-surfaces and on 3D porous scaffolds. Regardless of the medium-to-cell ratio, by day 7 cell viability was maintained at nearly 98%. On both 2D and 3D cultures, 0.04 µl per cell at seeding promoted greater cell proliferation than 0.08 and 0.16 µl per cell during the initial phase of culture. This trend was reversed on day 7, when the cells approached confluence. In the present study, 25 ml medium in the bioreactor, corresponding to 0.04 µl per cell initially, was considered to be optimal because cell proliferation was expected to be suppressed by fluid shear stress (Yamada et al., 2021a). It is of interest to note that the volume of culture medium influences not only cell growth but also osteogenic differentiation. Previous studies using osteoblastic cells showed that reduction of mineralization occurred in a medium volume-dependent manner: the more medium used, the less mineralization (Yoshimura et al., 2017). Furthermore, Schreivogel et al. reported that mechanical stimuli in their bioreactor increased the secretion of bone morphogenetic protein 2 by MSC but did not induce activation of downstream signaling in their original experimental configuration. This discrepancy was solved simply by reducing the volume of culture medium and increasing the number of cells, indicating that the excessive use of culture medium dilutes secreted factors and masks phenotypical amelioration (Schreivogel et al., 2019). On 3D polymeric scaffolds, high seeding density supports the osteogenic phenotype of BMSC and enhances bone regeneration after transplantation (Yassin et al., 2015). This may be convenient for perfusion systems where a large volume of medium is required to maintain continuous flow. Together, these results confirm the importance in dynamic cell culture systems, of determining the optimal seeding density and the minimum necessary medium volume, i.e., conditions which do not cause nutrient depletion but allow the cells to condition the medium by paracrine factors.

Humidity control is a key consideration during cell culture because medium condensation disturbs the osmotic balance, and the resulting high tonicity leads to cell dehydration (Triaud et al., 2003; Chi et al., 2020). This study demonstrated that medium perfusion without humidification concentrated medium components taking glucose concentration as an example, and the concentrated medium significantly affected cell viability and growth. The concept of developing tissue engineering bioreactors is mainly classified into systems installed in conventional incubators and stand-alone bioreactor, i.e., which requires an integrated incubation unit for environmental control (Li et al., 2014). However, unlike a standard incubator, maintaining humidity above 90% is not always agreeable in bioreactors because a humidified environment may cause malfunction or possibly irreparable damage to electrical components such as sensors, pumps, electric sockets and conducting wires from a long-term perspective. Admittedly, there seems a lack of consideration with regards to humidification control in previously developed systems. Furthermore, perfusion accelerates the evaporation ratio in a velocity-dependent manner (Handa et al., 1987; Sumino and Akiyama, 1987). The present study also disclosed notable medium loss during perfusion in the non-humidified condition, and this may potentially be detrimental to cell viability and growth. The placement of a water bath, as with a standard incubator, prevented evaporation as expected. However, aqueous droplets and moisture condensation were actually found on the surface of the bioreactor. As an alternative, an additional flask containing water was connected to the filter to humidify the local atmosphere contacting the culture medium. This measure had a comparable suppressive effect on evaporation, without increasing humidity inside the bioreactor. This procedure may be applicable to most bioreactor systems, to improve the culture environment under conditions in which a water bath may not be feasible.

Air bubble formation is a long-standing issue in fluidics (Lochovsky et al., 2012; Kang et al., 2014). It impedes or blocks fluid flow, and more importantly for bone tissue engineering, air bubbles entrapped in microporous scaffolds disrupt cell growth and migration to some extent, depending on cell type and the size/number of bubbles (Podichetty and Madihally, 2014; Bergemann et al., 2015). Bubbles are a determinant of protein denaturation (Faustino et al., 2009): a gas-liquid interface in form irreversibly alters the superorganization of protein molecules by absorbing and forming aggregates, which may result in loss of biological activity. This happens particularly to proteins with high surface activity (Clarkson et al., 1999). It is reported that approximately 10% of proteins in bovine serum albumin were denatured when the proteins were absorbed to and desorbed from air bubbles (Clarkson et al., 1999). The use of perfusion bioreactors for bone tissue engineering exacerbates conditions conducive to bubble formation. Culture medium is normally supplemented with serum/proteins as nutrient sources, acting as surfactants (Faustino et al., 2009). Surfactants lower the surface tension, facilitating the formation of bubbles in the presence of agitation and stabilizing them. Microporous scaffolds of synthetic polymers, which are preferred in bone tissue engineering for their mechanical strength, formability, biocompatibility and biodegradability, exacerbate the problem because of their high porosity and hydrophobicity (Gunatillake and Adhikari, 2003). When fluid flow encounters micropores, stirring may trigger bubble formation, particularly on hydrophobic materials, which absorb gasses and form thin air layers on the surfaces (Hanwright et al., 2005; Lee et al., 2005; Yu et al., 2017b; Yao et al., 2020). It has been shown that using a bubble trap effectively removes large bubbles from the circulation and prevents them from entering the culture chambers, but the trap neither prevents bubble formation nor removes bubbles trapped in the scaffold micropores (Li et al., 2014; Schuerlein et al., 2017). Alternatively, a complex tubing strategy is needed to control flow paths to isolate bubbles from the main stream (Bhaskar et al., 2018). Therefore, preventive measures of air bubble formation should be prioritized. In the present system, the formation of air bubbles was so extreme that rBMSC were severely affected. For total prevention, a simple yet rigorous step was taken: namely, elevation of the medium reservoir by 30 cm to apply approximately 20 mmHg (equivalent to 2.7 kPa) hydrostatic pressure onto the culture chamber. This was based on Henry’s law, which governs gas solubility in liquid: at a given temperature, gas solubility is proportional to static pressure (Kang et al., 2007). It was shown that a slight increase in static pressure decreased gas release in the culture chambers, which created a bubble-free environment. The degree of pressure required may depend on experimental settings, including material selection, scaffold geometry, and flow characteristics. Previous studies have suggested that BMSC undergo osteogenic differentiation under high static pressure ranging from 10 to 100 kPa (Huang et al., 2015; Zhao et al., 2015; Stavenschi et al., 2018). In the present setting, where 2.7 kPa was sufficient to eliminate bubbles completely, its effect on osteogenesis would be negligible although the present study did not evaluate the effect solely. Nevertheless, the strategy of supressing bubbles by applying a static pressure may interact favorably with osteogenic activity given that continuity is commonly observed in biological events.

Finally, the optimization of flow rate for the purpose of bone tissue engineering was explored by testing relatively low-level shear force. The reasons were twofold: not only is cell fate fine-tuned by mechanical stimuli, but also the results should be relevant to clinical translation. In other words, cells which are maintained in a perfusion bioreactor should be also supported during integration at the recipient site in the absence of the robust perfusion provided in bioreactors. In the present study, we tested a subphysiological level of perfusion, which reportedly initiated osteogenic differentiation on 2D surfaces (Coughlin and Niebur, 2012; Gao et al., 2014; Kim et al., 2014). rBMSC were sensitive enough to distinguish 0.8 ml/min (i.e., shear stress: maximum 13.1 mPa, mean 0.88 mPa, mode value 0.5–1 mPa), 1.6 ml/min (i.e., maximum 26.2 mPa, mean 1.76 mPa, mode value 1.0–1.5 mPa), and 3.2 ml/min (i.e., maximum 52.6 mPa, mean 3.51 mPa, mode value 2–3 mPa), and the cells responded differently. Even at the low levels of fluid stimuli, cell proliferation was either delayed or suppressed. Perfusion for 8 h at 0.8 ml/min was found to be compatible with the cell growth and viability, maintaining intact the morphology of the cells and cell-to-cell integration. The finding agrees with a previous report using an osteoblastic cell line, MC3T3-E1, in which shear stress above 1 mPa suppressed cell growth on a 3D scaffold (Cartmell et al., 2003).

In 2D perfusion settings, MSC as well as osteoblasts seem tolerant of high shear stress over 3 Pa and respond to the stimuli by upregulating the expression of osteogenic markers (McAllister et al., 2000; Jiang et al., 2002; Yourek et al., 2010; Mai et al., 2013; Lim et al., 2014; Liu et al., 2015; Yu et al., 2017c). However, the cells are reportedly more vulnerable to shear stress in 3D environment, and a sub-pascal level of shear stress sufficiently stimulates the osteogenicity without deteriorating general cell health (Porter et al., 2005; Gaspar et al., 2012). Previous studies using 3D dynamic culture systems showed that extremely low shear stress ranging from 5 to 10 mPa shear stress for 16 days increased the calcium deposition by rBMSC under the presence of osteogenic supplement (Sikavitsas et al., 2003; Sikavitsas et al., 2005). The promotion of osteogenesis by a low shear stress magnitude was also reported with human BMSC on various scaffold materials (Grayson et al., 2008; Li et al., 2009).

In our experimental setting, shear stress ranging from nearly 0 to up to 15 mPa (mode value 0.5–1.0 mPa) allowed rBMSC to upregulate the key transcription factors for osteogenesis, RUNX2 and Osterix, even in the absence of osteogenic chemical supplements. Osteogenic differentiation was confirmed by enhanced calcium deposition and ALP activity. Therefore, with the scaffold geometry, material selection, and cell type in the present study, it was concluded that this level of shear stress was optimal for balancing the induction of osteogenesis and the growth of rBMSC. Nevertheless, the optimal magnitude of fluid stimuli would differ according to cell types (e.g., species, donor sites, individual variations) and scaffold properties (e.g., micro-, and macro-geometry, surface chemistry, size). Biological responses may therefore differ, even if the same flow rate is applied. This underlines the importance of flow optimization and its challenges when in future clinical translation, scaffolds are custom-designed and loaded with patient-specific cells (Roseti et al., 2017).

## Conclusion

In bone tissue engineering, bioreactors are intended to support growth and targeted differentiation of stem/progenitor cells. There is a wide range of bioreactor systems in use, each with unique features. Moreover, dynamic cell culture inevitably involves parametric deviations from conventional static culture, which may mask or exaggerate effects of interest. As a previous study confirmed, exact comparative studies can probably be done only by using an identical “standardized” system under the same conditions (Israelowitz et al., 2012). However, some optimized parameters would be transferable to other systems and study designs. The present study explored some of basic but crucial optimization steps, namely the computational estimation of fluid force, the determination of culture medium volume, humidification, the strategy of air bubble suppression, and the identification of optimal fluid shear stress magnitude. The accurate estimation of fluid forces acts as a platform for understanding biological behaviors, while optimizing culture environmental factors contributes to stabilized and reproducible experiments. The thorough validation, optimization, and detailed description facilitate the further development of bioreactor applications in bone tissue engineering.

## Data Availability

The raw data supporting the conclusion of this article will be made available by the authors, without undue reservation.
