# Supplementary figures and images for: Optimization and Validation of a Custom-Designed Perfusion Bioreactor for Bone Tissue Engineering: Flow Assessment and Optimal Culture Environmental Conditions
Source: Front Bioeng Biotechnol. 2022 Mar 25;10:811942. doi: 10.3389/fbioe.2022.811942 (PMC8990132; doi:10.3389/fbioe.2022.811942)

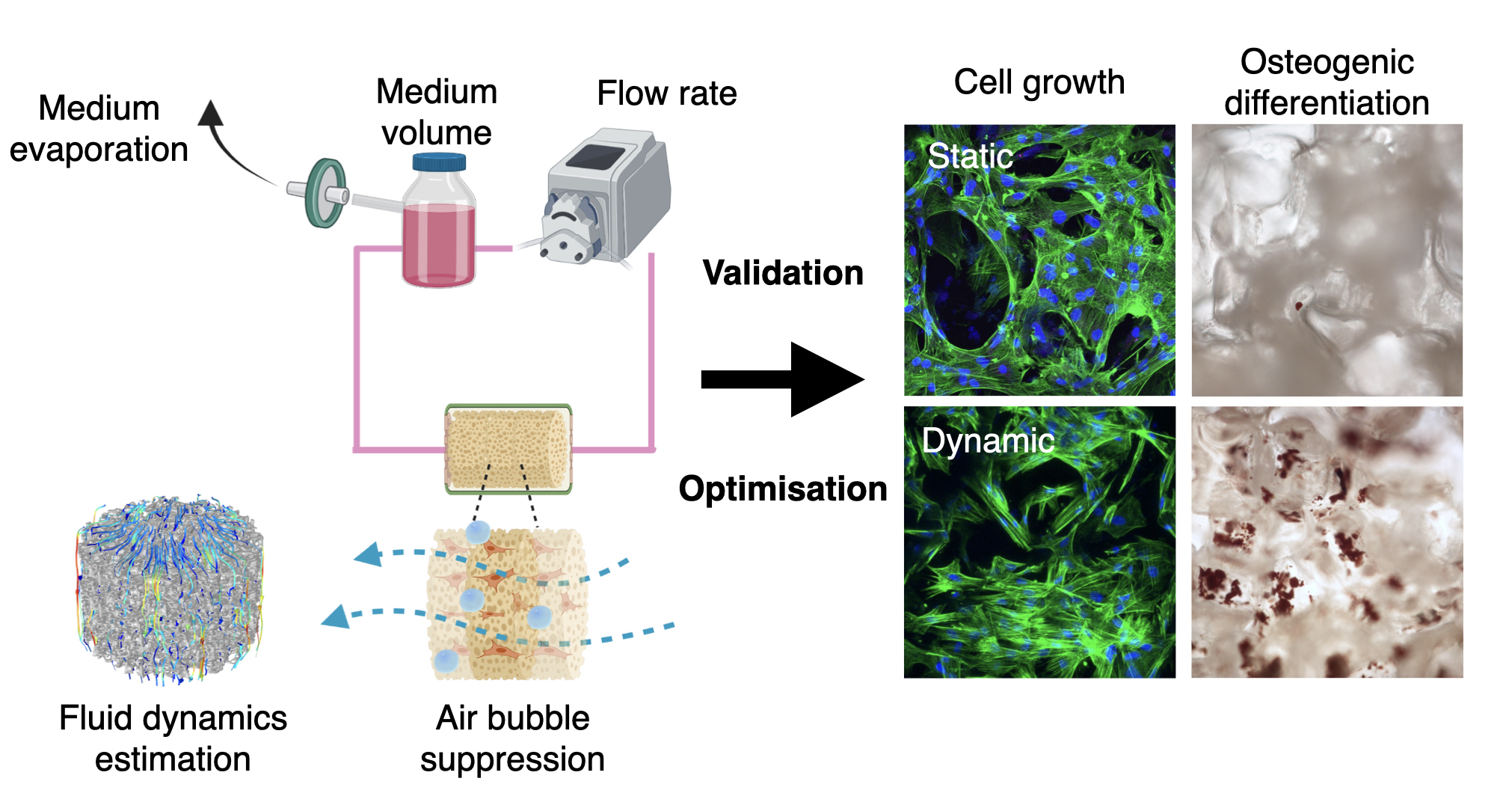

Supplement: Supplementary file 1 [file Image1.JPEG]
